# Posttranscriptional regulation by RNA-binding proteins during epithelial-to-mesenchymal transition

**DOI:** 10.1007/s00018-013-1379-0

**Published:** 2013-05-29

**Authors:** Luis A. Aparicio, Vanessa Abella, Manuel Valladares, Angélica Figueroa

**Affiliations:** 1 Translational Cancer Research Group, Instituto de Investigación Biomédica A Coruña (INIBIC), Complejo Hospitalario Universitario A Coruña (CHUAC), SERGAS, Xubias de Arriba 84, 15006 A Coruña, Spain; 2 Servizo de Oncología Médica, Complejo Hospitalario Universitario A Coruña (CHUAC), SERGAS, A Coruña, Spain

**Keywords:** Epithelial-to-mesenchymal transition, Tumor progression, Posttranscriptional regulation, RNA-binding proteins

## Abstract

Epithelial-to-mesenchymal transition (EMT), one of the crucial steps for carcinoma cells to acquire invasive capacity, results from the disruption of cell–cell contacts and the acquisition of a motile mesenchymal phenotype. Although the transcriptional events controlling EMT have been extensively studied, in recent years, several posttranscriptional mechanisms have emerged as critical in the regulation of EMT during tumor progression. In this review, we highlight the regulation of posttranscriptional events in EMT by RNA-binding proteins (RBPs). RBPs are responsible for controlling pre-mRNA splicing, capping, and polyadenylation, as well as mRNA export, turnover, localization, and translation. We discuss the most relevant aspects of RBPs controlling the metabolism of EMT-related mRNAs, and describe the implication of novel posttranscriptional mechanisms regulating EMT in response to different signaling pathways. Novel insight into posttranscriptional regulation of EMT by RBPs is uncovering new therapeutic targets in cancer invasion and metastasis.

## Introduction

Around 90 % of cancer-associated mortality is due to metastasis. Most of the human malignant tumors are carcinomas arising through transformations of epithelial cells. Cells that form the epithelial sheets in tissues are well polarized and tightly bound to each other by tight junctions, adherens junctions (AJs), desmosomes, and hemidesmosomes, forming a strong adhesive cell layer. Frequently, these structures strengthen physical interactions not only in normal epithelial cells but also in many benign carcinomas. AJs are major cell–cell junctions that mediate cell recognition, adhesion, morphogenesis, and tissue integrity. They are linked to the actin cytoskeleton, establishing molecular communication with other cell–cell junctions and cell–substratum adhesions, and they play a role not only in the organization and movement of the cells within the epithelium but also in the transmission of information to the interior of the cell. The best characterized member of AJs is E-cadherin, a transmembrane protein that contains an extracellular domain that forms homophilic interactions in a calcium-dependent manner and is responsible for cell–cell adhesion, and a cytoplasmic domain that is linked to the actin cytoskeleton through its interaction with several catenins such as α-catenin, β-catenin, and p120-catenin [1–5].

**Fig. 1:**
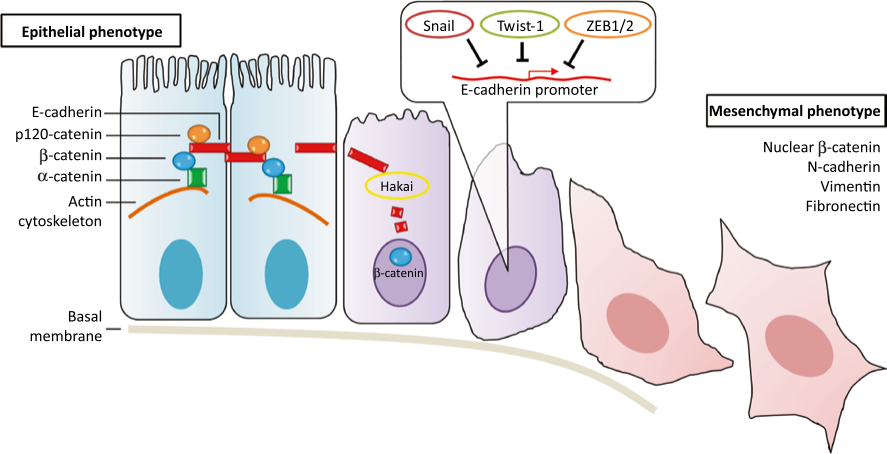
Epithelial-to-mesenchymal transition. Epithelial cells (*left*) are tightly interconnected by E-cadherin at adherent junctions. E-cadherin is linked to the actin cytoskeleton through different catenins. During EMT, E-cadherin can be downregulated by transcriptional repressors (such as Snail, Twist-1, and ZEB1/2), and by posttranslational regulators (such as Hakai). The loss of epithelial markers is accompanied by induction of vimentin, N-cadherin, and fibronectin, by increased levels of nuclear β-catenin, and by the promotion of a mesenchymal phenotype (*right*)

The epithelial-to-mesenchymal transition (EMT) process is an early step during carcinoma metastasis that resembles a highly conserved developmental program found in tissue remodeling during embryogenesis and in organ morphogenesis. EMT is characterized by the loss of the epithelial morphology and the acquisition of mesenchymal and motile characteristics, resulting from the loss of apical-basal polarity, the loss of cell–cell contacts, and the reorganization of the actin cytoskeleton [6–8] (Fig. 1). The EMT-associated downregulation of E-cadherin, a potent tumor suppressor, represents a hallmark of tumor progression [9]. Besides the downregulation of E-cadherin, several molecular markers characterize the EMT process, including the induction of several mesenchymal markers (such as N-cadherin and cadherin-11), the accumulation of β-catenin in the nucleus, the reorganization of the cytoskeleton, the switch from cytokeratins to vimentin, and changes in extracellular matrix (ECM) components and metalloproteinases [8, 10]. Numerous cytokines and autocrine growth factors, including TGFβ, have also been implicated in EMT [11–13]. In addition, molecular markers of EMT are associated with an increased capacity of cell migration and invasion, and with an elevated resistance to anoikis/apoptosis [7].

During EMT, E-cadherin is transcriptionally controlled by a number of well-established transcription factors, including Snail, Slug, Twist, ZEB1, and ZEB2, which associate with the E-cadherin promoter and induce its repression [14–16]. E-cadherin can also be controlled by posttranslational modifications, such as phosphorylation, glycosylation, and proteolysis [17, 18]. The first-described post-translational regulator of E-cadherin stability is Hakai, a protein that is also involved in controlling proliferation and other functions that influence tumor progression and disease [19–25] (Fig. 1).

Given the major impact of E-cadherin in cancer, the mechanisms involved in its inactivation in human cancers have been studied extensively. In recent years, posttranscriptional mechanisms that control E-cadherin expression have begun to emerge, bringing attention to the analysis of other posttranscriptional regulatory processes that influence EMT during tumor progression. In this review, we focus our attention on key posttranscriptional regulatory events controlled by RNA-binding proteins (RBPs) during EMT. We summarize the most relevant aspects of RBPs controlling key EMT-related mRNAs, as well as RBPs that modulate TGFβ-induced EMT [12]. This review paves the way for future investigation of novel posttranscriptional mechanisms regulating EMT in response to different stimuli, and identifies novel molecular candidates for drug design to treat cancer invasion and metastasis.

## Posttranscriptional regulation

The events that control gene expression at the posttranscriptional level include pre-mRNA splicing, polyadenylation, capping, and mRNA transport, turnover, storage, and translation. These posttranscriptional pathways function in precise coordination with transcriptional and posttranslational events to elicit a tight regulation of protein expression and function. Loss of these regulatory events impacts all areas of biology and medicine [26, 27]. In consequence, there has been a great deal of interest in understanding gene regulation on all levels, including posttranscriptionally, as they directly affect health and disease [28, 29].

In bacteria, genes are clustered into operons that are expressed in the form of polycistronic mRNAs; in the absence of a nuclear envelope, transcription and translation are coupled, allowing the efficient synthesis of gene products by ribosomes even before transcription has ended. By contrast, in eukaryotic cells the RNA is compartmentalized and transcription and translation are uncoupled. For several decades, most gene-regulation approaches focused mainly on transcription [30]; however, since the discovery of ribonucleoproteins (RNPs), interest in RNA biology has escalated rapidly [31–33]. This expansion was accelerated by the discovery of small regulatory RNAs, mainly microRNAs [34, 35]. Given that gene regulation in eukaryotes from transcription to translation is closely interconnected, many studies have suggested that posttranscriptional events involving multiple mRNAs are tightly coordinated as well [28, 36]. In light of this hypothesis, Tenenbaum and Keene proposed the “posttranscriptional operon model”, which postulates that RNA regulatory elements, frequently present in the untranslated regions (UTRs) of eukaryotic mRNAs, can interact with a single RBPs; in this manner, one RBP can jointly regulate expression of collections of mRNAs encoding proteins that are functionally related. Accordingly, the posttranscriptional control of mRNAs provides eukaryotic systems with the plasticity and effectiveness to respond to specific stimuli during different cellular processes [26, 37, 38].

**Fig. 2:**
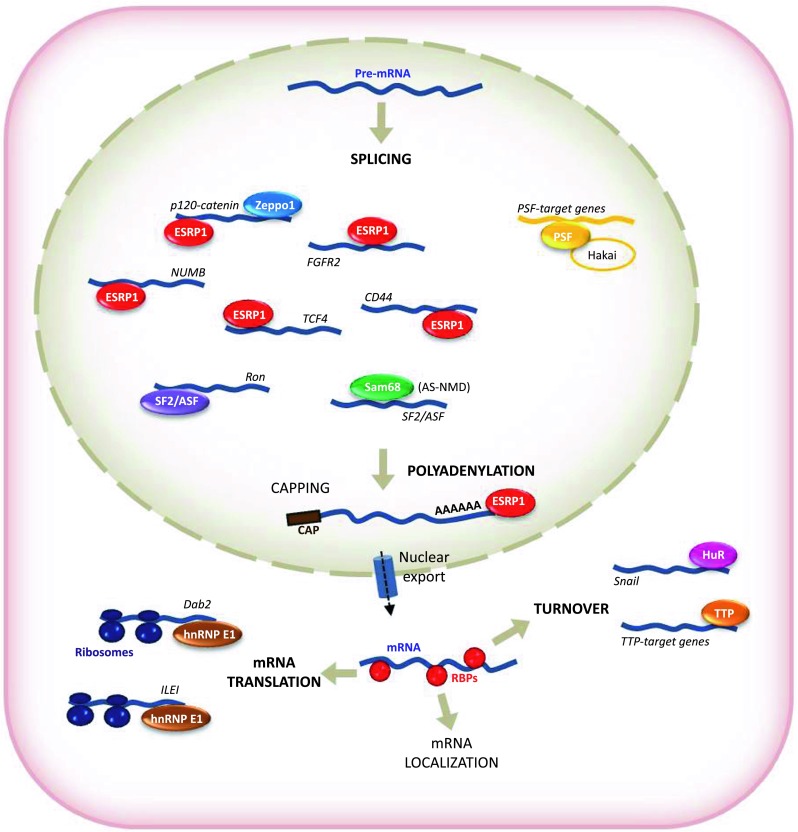
Schematic representation of posttranscriptionally regulated mRNAs by RBPs during EMT. RBPs involved in several posttranscriptional steps include ESRP1, Zeppo1, SF2/ASF (splicing), Sam68 (AS-NMD), ESRP (polyadenylation), hnRNPE1 (translation), TTP, HuR (mRNA turnover). PSF was first described as a splicing factor, although the specific posttranscriptional regulatory action on its mRNA targets remains unclear

Posttranscriptional regulatory events have been implicated in key molecular pathways in cancer development and metastasis. EMT-related gene expression programs are strongly influenced by posttranscriptional mechanisms governed by microRNAs, a family of small (~22 nt), and single-stranded and highly conserved non-coding RNAs that modulate gene expression in development and a vast array of diseases. miRNAs associate with mRNAs through partial complementarity and typically reduce their stability and/or translation status [28, 30–47]. MicroRNAs are also key regulators of tumorigenesis [48], including EMT, as discussed in numerous excellent reviews [49–55]. However, posttranscriptional regulation is also tightly controlled by RBPs that are involved in all aspects of mRNA metabolism [56–58]. RBPs bind to specific regions of the RNA (regulatory regions, *cis* elements) in mRNA UTRs. RBPs exist as complexes of proteins and pre-mRNA, named heterogeneous ribonucleoproteins (hnRNPs). RBPs are present in all living cells, and can bind to more than one mRNA with sequence specificity. Such interactions play an important role in regulating RNA localization and expression, and can coordinate the expression of mRNAs encoding functionally related proteins. In recent years, EMT has been shown to be robustly affected by posttranscriptional regulation processes. Although there is no evidence to date that regulated capping, export, or localization of the mRNA affects EMT, splicing, polyadenylation, stability, or mRNA translation critically influence EMT (Fig. 2; Table 1).

**Table 1:**
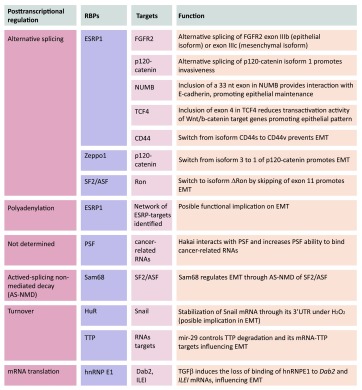
Summary of the described RBPs with roles in EMT

## EMT control by RBPs involved in alternative splicing and polyadenylation

Post-transcriptional regulation of gene expression by alternative splicing is important for normal physiology and disease [59] and was the first posttranscriptional mechanism linked to EMT [60]. It generates multiple mRNAs from a single transcript and is a major contributor to proteomic diversity and to the control of gene expression in complex organisms. Alternative splicing must be precisely regulated to ensure the expression of functionally different splice isoforms in a spatial and temporal manner. Conversely, deregulated alternative splicing may result in disease [61]. Changes in cellular phenotype, such as from the epithelial state to the mesenchymal state, is modulated at the level of alternative splicing by a number of factors.

### ESRPs

One of the first pieces of evidence associating a splicing factor to EMT was described for proteins ESRP 1 and 2. ESRPs were first identified as *e*pithelial *s*plicing *r*egulatory *p*roteins acting as specific regulators of *f*ibroblast *g*rowth *f*actor *r*eceptor-2 (FGFR2) splicing as well as other transcripts with epithelial-specific variants [62, 63]. By using a cell-based cDNA expression screening scheme, ESRP1 and ESRP2 were identified as regulators of FGFR2 splicing, showing that cell type-specific expression of epithelial and mesenchymal isoforms FGFR2 was achieved through the mutually exclusive exons IIIb and IIIc, respectively (Fig. 2). In keeping with the fact that ESRPs were found to be components of an epithelial gene signature, downregulation of ESRPs was associated with switches in splicing of several ESRP-regulated exons leading to changes in proteomic complexity during EMT [64].

Affymetrix human exon junction arrays (HJAY) (Affymetrix, Santa Clara, CA, USA) were used to identify changes in splicing profiles after ectopic overexpression of ESRP1 in MDA-MB231 mesenchymal cells and after knockdown of ESRP1 and ESRP2 in PNT2 epithelial cells. Microarray analysis of differential splicing plus (MADS+) computational pipeline was used to identify ESRP-regulated changes in splicing. HJAY arrays consist of eight tiled probes per probe set that target 315,137 exons and 260,488 exon–exon junctions, covering most human alternative splicing events supported by mRNA and/or EST evidence. The authors identified 417 and 540 changes in cassette exon splicing upon ectopic expression of ESRP1 and ESRP knockdown, respectively. With this approach, ESRP-regulated splicing network provided strong evidence that alternative splicing supplies an additional layer of gene regulation during EMT. Furthermore, the loss of this splicing program alone can induce some of the phenotypic changes that occur during EMT, supporting the hypotheses that the ESRP-regulated splicing program is an essential feature of the epithelial phenotype and that many changes in cell behavior associated with EMT are due to alterations in functions of proteins that undergo isoforms switching during this process. ESRPs are among the essential epithelium-specific genes which, like E-cadherin, can be downregulated directly or indirectly by mesenchymal transcription factors such as Twist, Snail/Slug, or Zeb1/2 in order to achieve a complete phenotypic cellular switch during EMT.

Changes in cell morphology and behavior through abrogation of an epithelial-splicing program suggests that the ESRPs regulate transcripts that, while expressed in both epithelial and mesenchymal cells, encode protein isoforms specific to each cell type with isoform-specific functions impacting on EMT. This impact is nicely illustrated by p120-catenin, a protein that switches isoforms and is extensively implicated in EMT. p120-catenin regulates Rho GTPases and promotes the invasiveness of E-cadherin-deficient cancer cells. Multiple p120 isoforms are expressed in cells via alternative splicing, and all of them are essential for HGF signaling to Rac1. However, only full-length p120 (isoform 1) promotes invasiveness mediated by reduced RhoA activity, both under basal conditions and following HGF treatment. All p120 isoforms can bind RhoA in vitro, via a central RhoA-binding site. However, only the cooperative binding of RhoA to the central p120 domain and to the alternatively spliced p120 N-terminus stabilizes RhoA binding and inhibits RhoA activity. In turn, increased expression of p120 isoform 1 is predictive of renal tumor micrometastasis and systemic progression following nephrectomy. ESRPs regulate the splicing of exons that generate different p120-catenin isoforms in epithelial and mesenchymal cells, promoting cell–cell adhesion in epithelial cells and cell migration and invasion in mesenchymal cells [62, 65] (Fig. 2). Numerous examples of ESRP-regulated transcripts encoding proteins related to EMT have been identified. Among them are several encoding proteins, such, for example, EPB41L5, SCRIB, MACF1 (ACF7), GIT2, LOXL2, FAT and PTPRM, implicated in the regulation of the actin cytoskeleton, cell–cell contacts, cell–matrix adhesion, migration, and polarity.

By using high-throughput screening (RNA-Seq), a complete spectrum of alternative splicing events regulated by ESRP1 and ESRP2 was examined. These analyses elucidated the global posttranscriptional regulation of splicing by ESRP, defining a network of ESRP-targets that encode proteins involved in pathways and the protein interaction networks that can be crucial during EMT in development and disease. By using deep-sequencing analysis of the transcriptomes in an in vitro model of EMT, global alternative splicing programs that alter splicing of key regulators of cell phenotype were discovered. The key regulators include proteins that control cell adhesion and cytoskeletal dynamics.

Several splicing factors, including ESRPs and members of the RBFOX, CELF, MBNL, and various hnRNP families, have been involved in EMT-associated splicing. Partial induction of the epithelial splicing program in mesenchymal cells via ectopic expression of ESRP1 or depletion of RBFOX2 (a splicing factor recently found to regulate subtype-specific splicing in breast cancer cells), conferred epithelial properties to mesenchymal cells, supporting a key role for alternative splicing during mesenchymal-to-epithelial transition. Multiple EMT-associated alternative splicing events were identified in breast cancer cell lines and in primary human breast cancer samples, where epithelial and mesenchymal splicing patterns were inversely correlated. It was proposed that this EMT-associated splicing signature could be used to detect EMT in primary human cancers with a potentially significant prognostic value [66, 67]. Among the ESRP targets identified in these studies are numerous examples of transcripts encoding proteins with functions involved in processes that underline the EMT such as regulation of actin-cytoskeleton, cell–cell and cell–matrix adhesion, migration, and cell polarity. One of the targets described is NUMB, which plays an essential function in the maintenance of cell polarity and cell–cell adhesion through the binding to N-terminal phosphotyrosine-binding (PTB) domain of E-cadherin. Abolishing this interaction during EMT leads to loss of cell–cell adhesion and to an increase in cell migration [68]. The NUMB-E-cadherin interaction is likely to be promoted by a 33-nt epithelial-specific exon in *NUMB*, which encodes an 11-amino-acid insert in this PTB-interacting domain. Isoforms that contain this insert are predominantly associated with the plasma membrane, whereas isoforms excluding these nucleotides are diffusely cytoplasmic [69].

Another example was described for β-catenin re-localization from adherens junctions to the nucleus, which occurs when E-cadherin is downregulated. In the nucleus, β-catenin associates with TCF/LEF transcription family members (including *TCF4*) inducing changes in gene expression associated to EMT [70, 71]. ESRPs promote inclusion of the exon 4 in *TCF4* (also known as *TCF7L2*) transcripts, whereas this exon is predominantly skipped in mesenchymal cells (Fig. 2). Isoforms that include exon 4 were recently shown to have reduced transactivation activity of several Wnt/β-catenin-target genes, suggesting that a switch towards the skipped isoform during EMT might further enhance the transcriptional activation of β-catenin-target genes upon its translocation to the nucleus [72]. These examples highlight several of the ESRP-regulated transcripts with EMT-related functions and also provide evidence that the resulting epithelial–mesenchymal isoforms have different and possibly opposing functions in the maintenance of cell adhesion versus cell migration. Most of the changes in splicing of ESRP-regulated exons have not been functionally characterized, however, several ESRP-regulated transcripts, such as NUMB, BIN1, ENAH, PALLD, or TCF4 [68, 72–76], are regulated by splicing and can generate isoform changes, impacting their functions that might be relevant as a result of ESRP downregulation during EMT (Fig. 2).

ESRP is also involved in the control of the alternative splicing of the *CD44* gene, also important during cancer progression. CD44 is a cell surface protein that modulates cellular signaling by forming complexes with various receptor tyrosine kinases [77, 78]. Alternative splicing of CD44 is also differentially regulated during EMT, by switching from the variable exon–containing CD44v isoforms to the standard isoform, CD44s. The switch to CD44s accelerates EMT and breast cancer progression (Fig. 2); accordingly, the mesenchymal CD44s isoform was found to be upregulated in advanced human breast tumors, supporting the notion that alternative splicing regulation is a critical mechanism in controlling EMT and cancer progression. ESRP1 is responsible for the switch in CD44 isoform expression, promoting the production of CD44v. *ESRP1* mRNA was drastically decreased during EMT, and correlated with the downregulation of CD44v and the upregulation of CD44s. Overexpression of ESRP1 in the Twist-inducible EMT system prevented the switch in CD44 isoform expression and blocked EMT. The acceleration of EMT seen after silencing ESRP1 was diminished when CD44 was depleted in the ESRP1-silenced cells, further confirming that ESRP1 negatively regulates EMT by preventing the shift in expression from CD44v to CD44s [79]. However, ESRP is not likely to be the only splicing regulator of CD44 affecting EMT and other splicing factors will likely emerge in the control of CD44 alternative splicing. Moreover, CD44s activates Akt, which results in an impaired expression of E-cadherin in TGF-β–induced mesenchymal MCF10A cells. The PI3 K/Akt signaling pathway has been shown to promote EMT, in part through the downregulation of E-cadherin [80]. CD44s expression increases cell survival and this effect is dependent on Akt signaling, while CD44v does not potentiate Akt activity or induce TGFβ-induced EMT. The link between CD44s, Akt signaling, and EMT, warrants future investigation to identify receptor tyrosine kinases that could interact with CD44s during EMT. A switch from CD44v in the epithelial primary tumors to CD44s in vivo was observed in a mouse model of breast tumor formation and recurrence [79]. In this model system, a statistically significant correlation between CD44s expression and tumor grade was found, with elevated N-cadherin mesenchymal marker levels, further supporting the essential role of CD44s during EMT.

### Zeppo1

Another important example of the consequence of an alternative splicing was described for p120-catenin and the splicing factor Zeppo1. p120 interacts with the cytoplasmic domain of E-cadherin at cell–cell contacts linking to the actin cytoskeleton and regulating cell adhesion. Localized in the cytoplasm and in the nucleus, p120 is expressed from multiple mRNA isoforms generated by alternative splicing, allowing translation to be initiated from four start codons and enabling the inclusion of four alternatively used exons affecting cadherin turnover and intracellular signaling [81–84]. p120 isoforms 1 and 3 are the most commonly expressed; isoform 1 is predominantly expressed in motile cells and in epithelial tumors and its presence predicts poor outcome in invasive breast cancer [85], while isoform 3 is the predominant isoform in epithelial cells, suggesting its role in EMT. In 2011, Zeppo1 was identified as a regulator of cell adhesion, migration, and proliferation of epithelial cells. In human breast cancer, an amplification of chromosome 8p11-12 that contains Zeppo1 was associated with increased proliferation and tumor grade and with reduced metastasis-free patient survival [86, 87]. Zeppo1 overexpression reduces cell–cell adhesion and increases migration and proliferation. Zeppo1 is a transcriptional repressor of E-cadherin promoter and induces p120 isoform 1 expression and the alteration of its localization, upon cell contact to extracellular matrix [88] (Fig. 2). Overexpression of Zeppo1 in cells grown on an ECM substrate causes preferential switch to isoform 1, while transcriptional repression of E-cadherin in combination with isoform switching from isoform 3 to 1 of p120 was described in MDCK cells overexpressing the EMT-inducing transcriptional repressors Snail, Slug, or E47 [89, 90]. The localization of p120 protein away from cell–cell contacts was also dependent on cell–ECM contact. In 2D Matrigel culture, Zeppo1 overexpression induced nuclear localization of isoform 3, while isoform 1 was restricted to the cell membrane and cytoplasm. In 3D culture, both isoforms were localized in the cytoplasm in migratory cells in contact with the ECM, but were localized to the cell membrane in cells in the body of the aggregates (away from the ECM). Moreover, the overexpression of Zeppo1 induced p120 switching from isoform 3 to 1 leading to an increased binding of p120 to RhoA, which in turn decreased RhoA activity. Given that the amplification of chromosome *8p11*-*12* in a mammary tumor cell leads to overexpression and increased activity of Zeppo1, E-cadherin expression is reduced with a consequent reduction in tumor cell adhesion. Loss of E-cadherin at the cell membrane reduces membrane-localized p120 and increases the pool of cytoplasmic p120 that interact with Rho GTPases. These results implicate Zeppo1 in the reduction of tumor cell adhesion and cell polarity and in the increase in tumor cell migration and proliferation, promoting an increase in tumor metastases [88].

### PSF

The RBP PSF (*p*olypyrimidine tract-binding protein-associated *s*plicing *f*actor) has also been reported to influence the expression of EMT-related proteins. PSF is a nuclear protein implicated in DNA transcription and repair, as well as in pre-mRNA splicing and RNA editing [91, 92]. It can interact with Hakai, which in turn can be crucial during EMT. As previously mentioned, Hakai is an E3 ubiquitin ligase that interacts with the cytoplasmic domain of E-cadherin in a tyrosine phosphorylation-dependent-manner, inducing the ubiquitination and endocytosis of the E-cadherin complex. Through the dynamic recycling of E-cadherin, Hakai can modulate cell adhesion and participate in the regulation of EMT during metastasis [19]. Hakai is also localized in the nucleus [20] and interacts with PSF, influencing PSF binding to specific target RNAs (Fig. 2). Indeed, when Hakai is overexpressed, PSF binding to mRNAs encoding cancer-related proteins increases, while knockdown of Hakai reduces the RNA-binding ability of PSF. In turn, important targets are induced such as those encoding proteins involved in tumor progression and angiogenesis (PAI-RBP1), tumor suppression (NF2, LKB1), cell–cell adhesion (α-catenin), or cytoskeleton dynamics (tubulin α-6). Thus, Hakai is proposed to modulate oncogenic phenotype by increasing PSF’s ability to bind RNAs to modulate cancer-related gene expression [20, 21]. However, it is still unclear whether Hakai influences the actions of PSF on splicing or other posttranscriptional steps on specific target RNAs.

### SF2/ASF and Sam68

EMT may also be triggered by the splicing factor and proto-oncogene SF2/ASF (splicing factor 2/alternative splicing factor) also known as splicing factor, arginine/serine-rich 1 or SFRS1. SF2/ASF regulates the alternative splicing of the *Ron* proto-oncogene, which encodes a tyrosine kinase receptor for the macrophage-stimulating protein involved in control of cell scattering and motility. ΔRon, a constitutively active isoform that confers increased motility to expressing cells, is generated through the skipping of exon 11. By controlling the production of ΔRon, SF2/ASF activates EMT leading to cell locomotion [93]. The splicing factor SF2/ASF is also controlled during in vitro EMT through the action of Sam68 by activated-splicing nonsense-mediated decay (AS-NMD) [94]. The mouse Src-associated protein Sam68 is similar to GLD-1, identified in *Caenorhabditis elegans*. Conserved KH domain is found within the larger GSG domain, suggesting a biochemical function for GLD-1 protein in binding RNA. Sam68 is a member of the STAR family of RNPs that links extracellular signals to RNA metabolism [95–97]. Alternative splicing can also affect RNA stability by inclusion of premature translation termination codons that activate the NMD pathway [98]. NMD is involved in quantitative posttranscriptional regulation of gene expression through specific alternative splicing-activated nonsense-mediated decay (AS-NMD) [99]. AS-NMD was recently shown to regulate expression of specific gene families. Alternative exons containing premature in-frame stop codons, or introns in the 3′UTRs, are particularly frequent in mammalian genes for splicing regulators, such as splicing regulatory factors and hnRNP [100, 101]. Most of these proteins can regulate in a feedback mechanism their own mRNA level by modulating AS-NMD and hence maintaining homeostatic levels of the protein [101].

The impact of SF2/ASF alternative splicing programs on EMT was studied in an in vitro model. SF2/ASF overexpression promotes skipping of *Ron* exon 11 and results in the production of ΔRon, a constitutively active isoform that confers invasive phenotype. EMT can be recapitulated in vitro by growing colon adenocarcinoma SW480 cells at different densities: at low density, sparse cells mimic the situation occurring at the invasive front of the tumor and display a mesenchymal phenotype, whereas at high density, the same cells switch to an epithelial phenotype. In this system, the level of splicing factor SF2/ASF is controlled during in vitro EMT through AS-NMD implicating Sam68 [94]. This regulation depends on diffusible factors expressed by epithelial cells through the ERK1/2 pathway and Sam68 that control SF/ASF transcripts and ΔRon mRNA production. In conclusion, cell growth conditions and external cues act through the ERK1/2 signaling pathway to modulate the activity of two splicing regulators, Sam68 and SF2/ASF, critically involved in EMT/MET at the posttranscriptional level in SW480 cells [94].

## EMT control by RNA-binding proteins involved in mRNA turnover and translation

In addition to alternative splicing, mRNA turnover and translation are key posttranscriptional events that control mammalian gene expression. The mechanisms determining mRNA turnover by RBPs generally involve recognition of specific RNA sequences.

### HuR

The ubiquitous RNA-binding protein HuR is one of the best-studied regulators of cytoplasmic mRNA fate. HuR is a ubiquitously expressed member of the embryonic lethal abnormal vision (ELAV) family of RNPs [102]. AU-rich elements (AREs), usually found in the 3′ untranslated region (UTR) of the mRNAs [103], are among the best-characterized mRNA determinants of stability and are selectively recognized by HuR. Through its posttranscriptional effect on specific mRNA targets, HuR can influence different cellular processes such as differentiation, proliferation, cancer, apoptosis, inflammation, and the stress response [104–115]. The Snail mRNA, which bears AREs in its 3′UTR, is one of the described HuR targets (Fig. 2). As previously reported, Snail is a transcriptional repressor of E-cadherin and plays a crucial role in the induction of EMT [14, 15]. HuR binding to the Snail 3′UTR increases following exposure to the oxidant hydrogen peroxide (H_2_O_2_), which like other reactive oxygen species, can trigger EMT in several cell types [116]. After lowering endogenous HuR levels, H_2_O_2_ reduces the levels and half-life of Snail mRNA, which in turn downregulates E-cadherin and enhances cell migration [117]. Thus, stabilization of Snail mRNA by HuR in response to H_2_O_2_ may facilitate EMT.

### Tristetraprolin

A member of the TIS11 family of RNPs, Tristetraprolin (TTP), also plays a critical role in regulating the expression of ARE-containing mRNAs. Through binding to ARE-containing mRNAs and targeting them for rapid degradation, TTP plays a role in limiting the expression of a number of critical genes eliciting anti-cancer effects [118, 119]. A role for TTP and mir-29 in epithelial polarity and metastasis has recently emerged [120]. This study employed the EpH4/EpRas/RasXT cell system, wherein parental EpH4 cells are non-tumorigenic murine mammary epithelial cells and show typical physiological responses to relevant growth factors and cytokines and EpRas cells, derived from EpH4 cells, are still epithelial but have become tumorigenic due to the constitutive overexpression of oncogenic H-Ras-V12. EpRas cells can undergo EMT and metastasize in response to transforming growth factor-β (TGF-β); the resulting cells (named RasXT), stably maintain their mesenchymal phenotype through autocrine TGF-β loops. Genome-wide messenger RNA (mRNA) profiling of these cells showed regulation of many genes involved in tumor progression and identified new genes relevant for EMT and metastasis in human patients. In this system, TTP was found to be strongly downregulated in metastasic RasXT relative to epithelial EpRas cells, while miR-29a was identified as one of the first miRNAs with the capacity to both interfere with and promote tumor progression, depending on the oncogenic context. miR-29a regulates the expression of TTP through its binding to two regions in the TTP 3′UTR and controls TTP during EMT from EpRas to RasXT cells. Overexpression of miR-29a suppressed TTP production, and thereby affected the levels of TTP target mRNAs (which also bear AREs in their 3′UTRs). Similarly, E-cadherin and vimentin levels correlated with the degree of miR-29 upregulation and TTP downregulation, epithelial polarity, and EMT (Fig. 2). In addition, miR-29a was able to promote metastatic growth in vivo, since miR-29a-overexpressing EpRas cells injected into the tail veins of nude mice led to an increase in the number of metastatic foci in the lung 14 days later. Finally, enhanced miR-29a and reduced TTP levels were detected in breast cancer patient samples, further supporting their relevance in carcinogenesis [120].

## Posttranscriptional regulation of EMT by growth factors

Posttranscriptional regulatory events have also been reported as critical regulators of TGFβ actions, playing an indispensable role in TGFβ-induced EMT and metastasis. hnRNP E (also known as PCBP1) was shown to influence the TGFβ-modulated expression of EMT-specific proteins, and EMT itself. Like other hnRNPs, hnRNP E1 participates in RNA transcription, pre-mRNA processing and maturation, and mRNA export [121]. Its implication in promoting tumor progression was first reported in studies focused on expression of phosphatase of regenerating liver (PRL)-3. High levels of PRL-3 did not correlate with transcript levels, but was instead linked to regulated translation. The 5′UTR of *PRL*-*3* mRNA possesses triple GCCCAG motifs capable of suppressing mRNA translation through its interaction with hnRNP E1, which retards the recruitment of *PRL*-*3* mRNA to polyribosomes. The knockdown of hnRNP E1 causes upregulation of PRL-3 translation, activation of AKT, and promotion of tumorigenesis. An inverse correlation between hnRNP E1 and PRL-3 protein levels was reported in several human cancers, suggesting that hnRNP E1-mediated PRL-3 regulation has physiological and clinical relevance [122]. hnRNP E1 was later described to be modulated in TGFβ-induced EMT and tumor metastasis and was found to affect translation of ILEI and Dab2 mRNAs. Using normal murine mammary gland epithelial (NMuMG) cells and mouse mammary EpRas cells as in vitro models for TGFβ-induced EMT, two candidate EMT genes were defined: Disabled-2 (Dab2) and interleukin-like EMT inducer (ILEI). Dab2 is a putative tumor suppressor gene, but modulates late stages of tumor progression by promoting EMT-dependent metastasis. *ILEI* belongs to the *FAM3A*-*D* gene family and was initially identified as a candidate gene for autosomal recessive nonsyndromic hearing loss locus 17 (*DFNB17*). ILEI was shown to be translationally upregulated during EMT in EpRas cells. In this system, Dab2 and ILEI are required, but not sufficient to induce EMT. TGFβ treatment increased expression of Dab2 and ILEI protein without a concomitant increase in the respective mRNAs; polysome release experiments confirmed that Dab2 and ILEI were translationally regulated in a TGFβ-dependent fashion and this effect mapped to a 33-nt-long region, named “BAT”, in the 3′UTR of ILEI and Dab2 mRNAs. Using protein extracts, hnRNP E1 and other proteins bound to BAT were identified by mass spectrometric analysis. TGFβ treatment triggered the dissociation of hnRNP E1 to both Dab2 and ILEI BAT elements. In vivo interaction studies revealed that although Dab2 and ILEI mRNAs were constitutively expressed, hnRNP E1 interacted with them only in unstimulated cells. Moreover, hnRNP E1 contains an Akt consensus phosphorylation site at Ser43; TGFβ-mediated phosphorylation of hnRNP E1 at this site was proposed as possible mechanism for loss of translational silencing following TGFβ treatment [123]. hnRNP E1 silenced mRNA targets at the level of translation elongation by acting on the eukaryotic elongation factor-1 A1 (eEF1A1). Modulation of the steady-state levels of hnRNP E1 or its posttranslational modification repressed translation, tumorigenesis, and cancer metastasis [123–125]. In sum, hnRNP E1 may regulate a cohort of EMT and metastasis mRNA transcripts, although only *Dab2* and *ILEI* were identified thus far [124, 126].

As mentioned above, a variety of high-throughput analyses of gene expression support the notion fact that the transcriptome does not mirror with the proteome, underscoring the importance of translational regulation in the control of protein expression programs [127]. A cohort of translationally regulated mRNAs was identified as being induced during TGFβ-mediated EMT by using a genome-wide approach that combined mRNA expression profiling and RIP-Chip analysis [128]. In this analysis, a RBP of interest was immunoprecipitated, and the associated mRNAs were isolated and subsequently identified by microarray analysis; the subset of bound mRNAs was then used to gain information on particular regulatory pathways [129, 130]. Similar to *Dab2* and *ILEI* mRNAs, other mRNAs translationally silenced by hnRNP E1 were expected to contribute in TGFβ-induced EMT; thus, an approach combining polysome profiling and hnRNP E1 RIP-Chip analyses was performed. In silico analysis identified several other mRNAs that harbor the BAT structural element in the 3′UTR. Select transcripts were then identified, which were posttranscriptionally upregulated by TGFβ and correlated with an induction of the EMT phenotype [128]. This cohort of mRNAs may represent new TGFβ-responsive and hnRNP E1-mediated regulon, operating at a post-transcriptional level to mediate TGFβ-induced EMT in a tightly regulated fashion.

As mentioned above, among the well-characterized genes regulated by tissue-specific alternative splicing are FGFRs. Functional FGFRs are encoded by four genes (FGFR1–FGFR4), and the receptors consist of three extracellular immunoglobulin domains (Ig-I, Ig-II, and Ig-III), a single transmembrane domain, and a cytoplasmic tyrosine kinase domain [131]. The alternative splicing of FGFR2 in EMT was first discovered in rat bladder carcinoma in 1994 [132]. Since then, many reports have analyzed the molecular mechanism that governs this process. ESRPs 1 and 2 promote the splicing of exon IIIb and silence the splicing of exon IIIc of FGFR2, leading to the expression of proteins characteristic of the epithelium [62, 63]. TGF-β induces isoform switching from IIIb to IIIc of the FGFRs by alternative splicing during EMT (Fig. 2), which enables cells to become sensitive to FGF-2. Therefore, in TGF-β-treated cells, FGF-2 enhances EMT. TGF-β-induced EMT and long-term exposure to TGF-β triggered the epithelial-to-myofibroblastic transition (EMyoT) by inactivating the MEK-Erk pathway. During the EMT process, TGF-β induced isoform switching of FGFRs, causing the cells to become sensitive to FGF-2. Addition of FGF-2 to TGF-β-treated cells perturbed EMyoT by reactivating the MEK-Erk pathway and subsequently enhanced EMT through the formation of MEK-Erk-dependent complexes of the transcription factor δEF1/ZEB1 with its transcriptional corepressor CtBP1. Consequently, normal epithelial cells that have undergone EMT as a result of combined TGF-β and FGF-2 stimulation promoted the invasion of cancer cells. Thus, TGF-β and FGF-2 may cooperate to regulate EMT in various kinds of cells during cancer progression [133].

In addition to FGFR2, TGF-β regulates other alternative splicing events. By microarray analysis or exons, TGF-β was found to induce broad alteration of splicing patterns by downregulating ESRPs. Indeed, after TGF-β treatment, the expression of δEF1 family proteins (δEF1 and SIP1) increases while the expression of ESRP decreases. δEF1 and SIP1 each potently repressed ESRP2 transcription; silencing of both δEF1 and SIP1, but not either alone, abolished the TGF-β-induced ESRP repression. The expression profiles of ESRPs were inversely related to those of δEF1 and SIP in human breast cancer cell lines and primary tumors. Further, overexpression of ESRPs in TGF-β-treated cells inhibits the conversion of alternative splicing pattern of epithelial types into those of mesenchymal types, accompanied by the downregulation of E-cadherin expression [134], resulting in the restoration of the epithelial splicing profiles as well as attenuation of certain phenotypes of EMT. Elevated expression of δEF1 and SIP1 appears to correlate with aggressive phenotypes and poor prognosis of cancer patients, probably due to the invasive and metastatic properties of tumor cells via EMT [134]. Most cell lines with high δEF1 and SIP1 expression levels and low ESRP expression levels were categorized as being of the “basal-like” subtype of breast cancer, while most cell lines with low δEF1 and SIP1 and high ESRPs were categorized as being of the “luminal” subtype of breast cancer, although the levels of the EMT-regulators have not been characterized in either of these subtypes, [135, 136]. Taken together, the δEF1 family of proteins represses ESRP expression to regulate alternative splicing during TGF-β-induced EMT and the progression of breast cancer.

## Therapeutic opportunities

Given the great complexity displayed by human tumors not only among patients but also within a given tumor (in different areas or stages), molecular oncologists are switching their focus from the identification of irreversible mechanisms of cancer initiation and progression (such as mutations or amplifications) to the discovery of mechanisms (including transcriptional and posttranscriptional mechanisms) that can reverse cancer-associated gene expression programs. During the invasive phase of metastasis, a carcinoma cell activates EMT programs by different regulatory pathways. It is increasingly apparent that EMT and tumorigenesis are mediated by the interplay of transcriptional and posttranscriptional programs. This review has highlighted gene expression programs controlled at the level of alternative splicing, stability, and translation that govern EMT. As our understanding of the posttranscriptional events during EMT expands, it is becoming increasingly evident that RBPs are attractive therapeutic targets. Incorporating EMT-relevant RBPs into routine pathological exams could also help as prognostic markers as well as to identify resistance to specific chemotherapies. In this direction, the levels of select transcripts that are posttranscriptionally upregulated by TGFβ correlate with induction of the EMT phenotype. Further analysis of the proteins encoded by these targets will contribute to a better comprehension of the cancerous phenotype and provide more targeted tools for therapy. We anticipate that a more complete understanding of posttranscriptional gene regulation during EMT will help to identify effective new targets in cancer prevention, diagnosis, prognosis, and therapy.

## Abbreviations

AJs: Adherens junctions
AREs: AU-rich elements
AS-NMD: Activated-splicing nonsense-mediated decay
BAT: TGF-Beta-activated translation
CD44: Cluster of differentiation 44
CD44s: Cluster of differentiation 44 standard isoform
CD44v: Cluster of differentiation 44 variable isoform
ECM: Extracellular matrix
eEF1A1: Eukaryotic elongation factor-1 A1
ELAV: Embryonic lethal abnormal vision
EMT: Epithelial-to-mesenchymal transition
ENAH: Enable homolog
ESRPs: Epithelial splicing regulatory proteins
FGF2: Fibroblast growth factor 2
FGFR2: Fibroblast growth factor receptor 2
HnRNPs: Heterogeneous ribonucleoprotein particles
HuR: Human antigen R
ILEI: Interleukin-like EMT inducer
LKB1: Liver kinase B1
NF2: Neurofibromatosis type II tumor suppression protein
NMD: Nonsense-mediated mRNA decay
PAI-RBP1: Plasminogen activator inhibitor 1-RNA binding protein
PCBP1: Poly(rC)-binding protein 1
PI3K: Phosphatidylinositide 3-kinase
PRL-3: Phosphatase of regenerating liver 3
PSF: Polypyrimidine tract-binding protein-associated splicing factor
PTB: Phosphotyrosine-binding
RBPs: RNA-binding proteins
RIP: Chip RNA-binding protein immunoprecipitation-microarray
RNPs: Ribonucleoproteins
ROS: Reactive oxygen species
SF2/ASF: Alternative splicing factor/splicing factor 2
SIP1: Smad-interacting protein 1
TCF/LEF: Transcription factor/lymphoid enhancer factor
TCF4: Transcription factor 4
TCF7L2: Transcription factor 7-like 2
TGF-β: Transforming growth factor beta
TIS11: TPA-inducible sequence 11
TTP: Tristetraprolin
UTRs: Unstranslated regions
δEF1: δ-crystallin enhancer factor 1
